# Efficacy and safety of nebivolol in Korean patients with hypertension by age and sex: a subanalysis from the BENEFIT-KOREA study

**DOI:** 10.1186/s40885-021-00165-3

**Published:** 2021-03-15

**Authors:** Kyoung Im Cho, Dong Woon Jeon, Hyo Seung Ahn, Dong Kyu Jin, Hyun Sang Lee, Jong-Young Lee, Hong-Seok Lim, Athanasios J. Manolis, Seung-Woon Rha, Sang Won Park

**Affiliations:** 1 Division of Cardiology, Kosin University Gospel Hospital, Busan, Republic of Korea; 2 Division of Cardiology, National Health Insurance Service Ilsan Hospital, Goyang, Republic of Korea; 3 Division of Cardiology, Department of Internal Medicine, Sahmyook Medical Center, Seoul, Republic of Korea; 4 Division of Cardiology, Department of Internal Medicine, Soonchunhyang University Cheonan Hospital, Cheonan, Republic of Korea; 5 Department of Cardiology, CHA Gumi Medical Center, Gumi, Republic of Korea; 6 Division of Cardiology, Department of Internal Medicine, Kangbuk Samsung Hospital, Sungkyunkwan University School of Medicine, Suwon, Republic of Korea; 7 Department of Cardiology, Ajou University School of Medicine, Suwon, Republic of Korea; 8 Cardiology Department, Asklepeion General Hospital, Athens, Greece; 9 Cardiovascular Center, Korea University Guro Hospital, Seoul, Republic of Korea; 10 A. Menarini Korea Ltd., Seoul, Republic of Korea

**Keywords:** Essential hypertension, Nebivolol, Asian, Monotherapy, Combination therapy, Add-on therapy, Age, Sex

## Abstract

**Background:**

BENEFIT-KOREA (BEnefits after 24 weeks of NEbivolol administration For essential hypertensIon patients wiTh various comorbidities and treatment environments in Korea) study, an observational study in South Korea, demonstrated the efficacy and safety of nebivolol in Asian patients with essential hypertension with and without comorbidities in real-world settings. We present a subanalysis of the efficacy and safety of nebivolol across age and sex in the BENEFIT-KOREA cohort.

**Methods:**

Adult South Korean patients with essential hypertension participated in the prospective, single-arm, open, observational BENEFIT-KOREA study; 3011 patients received nebivolol as monotherapy or add-on therapy. Changes in systolic blood pressure (SBP) and diastolic blood pressure (DBP), and pulse rate at 12 and 24 weeks were evaluated. Participants were divided into three age groups—young males and females: < 50 years; middle-aged males and females: ≥50 years to < 70 years; and older males and females: ≥70 years.

**Results:**

The mean age of study participants was 63.5 ± 12.9 years; majority were between 50 and 69 years of age and 40.4% were females. A significant decrease was observed in mean SBP, DBP, and pulse rate from baseline at 12 and 24 weeks in males and females across all age groups analyzed (all *P* < 0.001 vs. baseline), with no significant difference in mean reduction in SBP and DBP from baseline between sex within the age groups. Majority of reported adverse events were mild. The incidence of adverse events was lower in young participants versus middle-aged and older participants.

**Conclusions:**

Our subanalysis from the real-world BENEFIT-KOREA study in Asian patients with essential hypertension demonstrated the efficacy and safety of once-daily nebivolol across age groups with no between-sex differences.

**Trial registration:**

Name of the registry: clinicaltrials.gov. Trial registration number: NCT03847350. Date of registration: February 20, 2019 retrospectively registered.

**Supplementary Information:**

The online version contains supplementary material available at 10.1186/s40885-021-00165-3.

## Background

Hypertension, the most common modifiable risk factor for all-cause mortality and morbidity worldwide, is a widespread public health challenge; approximately 1 billion people or 26% of the world population suffers from hypertension [1, 2]. The prevalence of hypertension is known to differ by age, sex, and other factors [3, 4] and it increases with advancing age [5, 6]; ~ 60% older adults become hypertensive [7].

Several studies have shown a higher prevalence of hypertension in males < 65 years of age compared with females of the same age group; however, in the sixth decade of life and beyond, the prevalence of hypertension becomes higher in females than in males [4, 8–11]. Similar data has been reported in Korean adults. In the Korean National Health and Nutrition Examination Survey (KNHANES) 2016, the prevalence of hypertension in adults between the age of 60–69 years and those ≥70 years was 51 and 69%, respectively [12]. The prevalence was higher in males (56%) than in females (46%) until the age of 60 years, but lower in males (64%) than in females (72%) after the age of 70 years [12]. Korean females > 60 years of age were more likely to have hypertension and less likely to maintain hypertension control than males in the same age group [3].

Observed sex-based differences in hypertension could be attributed to biological and behavioral differences between the sexes [13]. For instance, the sympathetic nervous system has a dynamic role in blood pressure (BP) maintenance and in the pathogenesis of hypertension [14, 15]. It changes with age and may have different extent of contribution for hypertension development in males and females [14, 15]. Despite sex differences in hypertension, the treatment guidelines for hypertension do not differ by sex [4].

Nebivolol is a vasodilatory β1-adrenergic receptor antagonist which has been reported to be efficacious and well tolerated for achieving BP control in patients with hypertension [16]. The BENEFIT-KOREA (BEnefits after 24 weeks of NEbivolol administration For essential hypertensIon patients wiTh various comorbidities and treatment environments in Korea) study, a non-comparative, non-controlled, prospective, single-arm, multicenter, observational study conducted at 66 sites in South Korea, demonstrated the efficacy and safety of nebivolol in Asian patients with essential hypertension with and without comorbidities in a real-world setting [17]. In this manuscript, we report the results of a subanalysis of the efficacy and safety of nebivolol across age and sex in the BENEFIT-KOREA cohort.

## Methods

Details of the study design and methodology are available in the primary manuscript of the BENEFIT-Korea study [17]. The study was conducted in accordance with the ethical principles that have their origins in the Declaration of Helsinki. All enrolled patients provided written informed consent prior to undergoing any study-related procedure. The study protocol and relevant documentation were approved by Institutional Review Board/independent ethics committee(s).

In brief, the BENEFIT-KOREA study was an open, non-comparative, non-controlled, prospective, single-arm, multicenter observational study which enrolled male and female patients ≥19 years diagnosed with essential hypertension. These patients could be newly diagnosed with hypertension and not receiving any anti-hypertensives, or previously diagnosed and receiving other anti-hypertensives, making a switch to nebivolol as combination or add-on therapy. The visit schedule is outlined in Table 1.

**Table 1:** Study visit schedule: BENEFIT-Korea study

Variable	Visit 1 (baseline)	Visit 2 (follow-up)	Visit 3 (follow-up/last visit)
Schedule (wk)	0	12 ± 2^a^	24 ± 2 ^a^
Informed consent form	√		
Inclusion/exclusion criteria	√		
Patient demographics	√		
Height/weight/waist size^b^	√	√	√
History of previous anti-hypertensive drugs	√		
Medical history^c^	√		
Administrative status of nebivolol	√	√	√
Concomitant medications	√	√	√
Laboratory test^d^	√	√	√
Blood pressure measure^e^	√	√	√
Safety assessment		√	√

^a^Patients were allowed ±2 weeks period from the designated schedules for follow-up visits. ^b^Height was measured only at baseline (height, weight, and waist size) was recorded up to one decimal. ^c^Past medical history and present medical history within 6 months from baseline were recorded. In case a medical history was collected after baseline, it was recorded in the safety analysis set. ^d^Glucose (HbA1c, fasting blood sugar), total cholesterol, triglyceride, high density lipoprotein cholesterol, low density lipoprotein cholesterol, and other laboratory tests, when done, were recorded. Of the laboratory test results collected, the values pointing to adverse events were recorded in the safety analysis set. ^e^Blood pressure was measured when subjects were stable - mean seated trough cuff blood pressure was measured

BP was measured based on guidelines from the Korean Society of Hypertension [18] and was in accordance with the European Society of Cardiology/European Society of Hypertension (ESC/ESH) guidelines. All participating centers were checked for compliance and the settings for BP measurement defined in the protocol at the initiation meeting. BP was measured when patients were in stable state with 5 min rest. The mean seated cuff BP was measured twice within 1-min interval using upper arm sphygmomanometer; either manual or automated device was permitted. BP measurements were recorded and presented as an average of two measurements. Pulse rate reading generated by automated device or pulse rate measured for 15 s before manual measurement multiplied by four were regarded as the pulse rate per minute. Site feasibility was checked and confirmed before study initiation to ensure study compliance.

Primary and secondary end points of the BENEFIT-KOREA study have been presented by Lee et al. [18]. In this manuscript, we present the results of a subanalysis of the participants for change in systolic BP (SBP) and diastolic BP (DBP) and pulse rate after 12 and 24 weeks of nebivolol treatment based on age and sex.

The safety set was defined as all participants who were administered nebivolol and underwent follow-up at least once during the study period. Efficacy parameters were analyzed in the efficacy set defined as all participants from the safety set who also had efficacy assessment data at 12 or 24 weeks. All statistical analyses were performed using SAS ver. 9.4 (SAS Institute Inc., Cary, NC, USA). Quantitative data were statistically analyzed using paired t-test. Safety endpoints of treatment emergent adverse events were assessed using the Medical Dictionary for Regulatory Activities (MedDRA) standardized terms.

## Results

### Baseline demographics and clinical characteristics

Of the 3250 participants enrolled in the data from 66 sites across South Korea, 3140 were included in the safety set. The efficacy set included 3011 participants, 129 participants whose SBP and DBP were not recorded at baseline or at 12 (±2 weeks) or 24 weeks (±2 weeks) were excluded from the safety set.

**Fig. 1:**
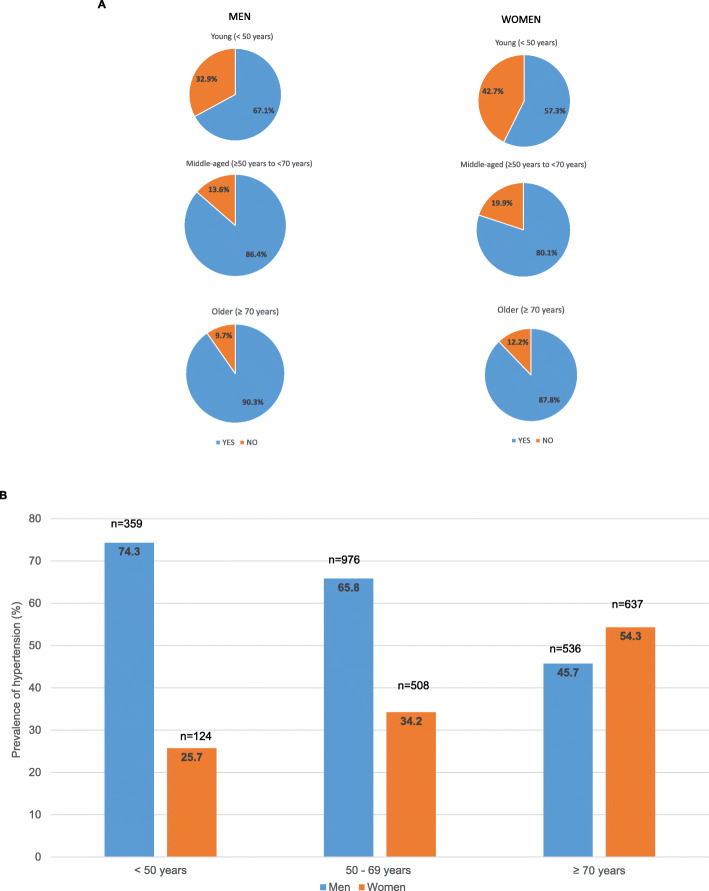
**a** Concomitant medications (other than anti-hypertensives) by age and sex in the safety population in the BENEFIT-Korea study; **b** Prevalence of hypertension by sex and age in the safety population in the BENEFIT-Korea study

Baseline demographics and the clinical characteristics of the safety population are summarized in Table 2. The mean age of study participants was 63.5 ± 12.9 years; majority of the participants were between 50 and 69 years of age and 40.4% were females. Cardiocerebrovascular risk factors were present in a higher proportion of males (97.5%) versus females (93.9%) and more males were current smokers compared with females (24.7% vs. 1.8%) (Table 2). The use of concomitant medications other than anti-hypertensives increased with age in both males and females (Fig. 1a).

**Table 2:** Baseline patient demographics: safety population BENEFIT-Korea study

Variable	Male (*n* = 1871)	Female (*n* = 1269)	Total (*n* = 3140)
Age (yr)			
< 50	359 (11.4)	124 (4.0)	483 (15.4)
50–69	976 (31.1)	508 (16.2)	1484 (47.3)
≥ 70	536 (17.1)	637 (20.3)	1173 (37.4)
Height (cm)^a^	168.7 ± 6.7	154.2 ± 6.1	162.7 ± 9.6
Body weight (kg)^b^	73.4 ± 12.5	60.8 ± 10.2	68.1 ± 13.2
Waist circumference (cm)^c^	90.0 ± 9.0	84.4 ± 9.6	87.7 ± 9.6
BMI (kg/m^2^)^d^	25.7 ± 3.5	25.5 ± 4.0	25.6 ± 3.8
Cardiocerebrovascular risk factors present	1825 (97.5)	1192 (93.9)	3017 (96.1)
Male ≥45 yr, female ≥55 yr	1643 (90.0)	1064 (89.3)	2707 (89.7)
Current smoker	451 (24.7)	21 (1.8)	472 (15.6)
BMI ≥25 kg/m^2^, or waist circumference > 90 cm (male) or > 80 cm (female)	673 (36.9)	455 (38.2)	1128 (37.4)
Dyslipidemia	911 (49.9)	613 (51.4)	1524 (50.5)
Impaired fasting glucose or impaired glucose tolerance	42 (2.3)	15 (1.3)	57 (1.9)
Family history of early cardiocerebrovascular disease (male, < 55 yr, female < 65 yr)	84 (4.6)	69 (5.8)	153 (5.1)
Diabetes mellitus	549 (30.1)	323 (27.1)	872 (28.9)
Medical history present	1667 (89.1)	1131 (89.1)	2798 (89.1)
Diseases of circulatory system	1234 (74.0)	773 (68.4)	2007 (71.7)
Endocrine, nutritional and metabolic diseases	1045 (62.7)	713 (63.0)	1758 (62.8)
Diseases of genitourinary system	276 (16.6)	123 (10.9)	399 (14.3)
Diseases of digestive system	138 (8.3)	140 (12.4)	278 (9.9)
Diseases of musculoskeletal system and connective tissue	98 (5.9)	111 (9.8)	209 (7.5)
Use of concomitant treatment with anti-hypertensives with nebivolol	1460 (78.0)	984 (77.5)	2444 (77.8)
Calcium antagonists	816 (55.9)	512 (52.0)	1328 (54.3)
Angiotensin II receptor antagonists	724 (49.6)	583 (59.3)	1307 (53.5)
Diuretics	299 (20.5)	279 (28.4)	578 (23.7)
ACE inhibitors	183 (12.5)	42 (4.3)	225 (9.2)
Alpha-blockers	24 (1.6)	9 (0.9)	33 (1.4)
Not available	10 (0.7)	2 (0.2)	12 (0.5)

Values are presented as number (%) or mean ± standard deviation

*P*-value (t-test) for the difference between male and female was significant (*P* < 0.001) for height, weight, and waist circumference

*BMI* body mass index, *ACE* angiotensin-converting enzyme

^a^Height measurement: male (1217), female (867). ^b^ Body weight measurement: male (1212), female (868). ^c^ Waist circumference: male (292), female (208). ^d^BMI: male (1174), female (846)

For the age and sex analysis, the participants were divided into three age groups—young males and females, < 50 years; middle-aged males and females, ≥50 years to < 70 years; and older males and females, ≥70 years. The categorization of older adults is in line with the SENIORS study population where the effect of nebivolol was assessed in elderly patients (≥70 years) with heart failure [19].

The prevalence of hypertension in the BENEFIT-KOREA study population is illustrated in Fig. 1b; the prevalence of hypertension increased in females with increasing age and was higher in females than in males in the older age group.

The total dose of nebivolol was significantly lower in females compared with males (*P* < 0.001) and in older participants compared with middle-aged and young participants (< 50 years, *P* < 0.001; 50–69 years, *P* < 0.001) (Table 3).

**Table 3:** Average daily dose and total dose of nebivolol in the safety population in BENEFIT-Korea study

Dose (mg)	Male (*n* = 1871)	Female (*n* = 1269)	Total (*n* = 3140)	*P*-value
Average daily dose of nebivolol	4.5 ± 0.99	4.4 ± 1.1	4.5 ± 1.0	
Total dose of nebivolol	792.1 ± 269.4	747.3 ± 290.1	774.0 ± 278.7	< 0.001^a^
Total dose of nebivolol by age (yr)				
< 50	–	–	818.5 ± 258.8	0.44^b^
50–69	**–**	–	800.9 ± 270.8	< 0.001^c^
≥ 70	–	–	721.6 ± 288.6	< 0.001^d^
Average daily dose of nebivolol by age (yr)				
< 50	–	–	4.8 ± 0.6	
50–69	–	–	4.6 ± 1.0	
≥ 70	–	–	4.2 ± 1.2	

Values are presented as mean ± standard deviation. Average daily dose of nebivolol = total dose of nebivolol / total treatment period; total dose of nebivolol = total dose of nebivolol X treatment period

^a^Wilcoxon rank sum test; comparison between males and females. ^b^Wilcoxon rank sum test, comparison between < 50 years and 50–69 years. ^c^Wilcoxon rank sum test; comparison between 50 and 69 years and ≥ 70 years. ^d^Wilcoxon rank sum test; comparison between < 50 years and ≥ 70 years

### Subanalysis outcomes

**Fig. 2:**
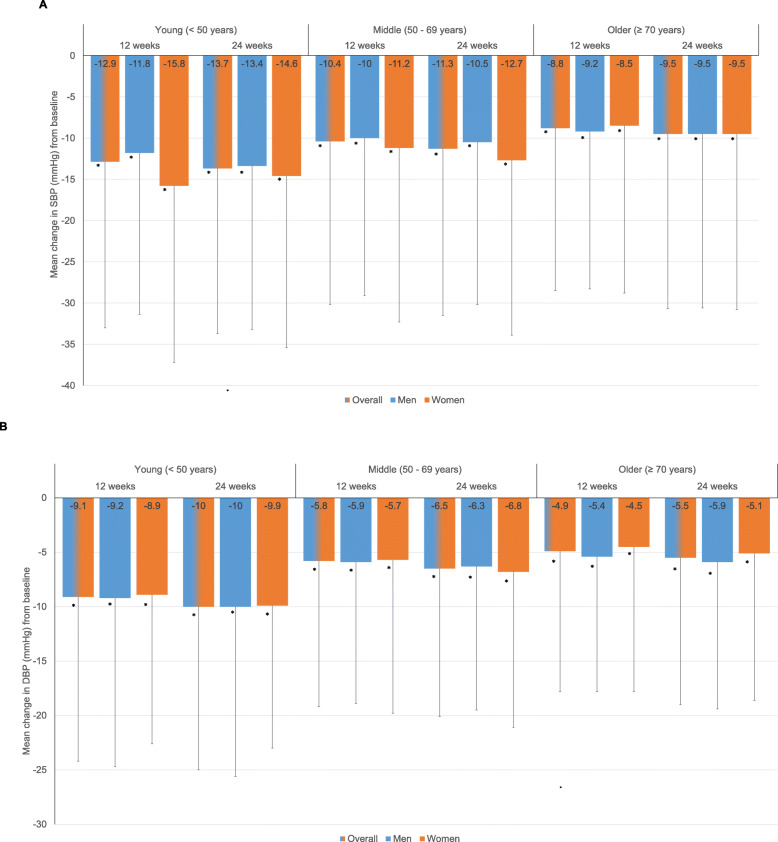
Mean change from baseline in (**a**) systolic blood pressure (SBP), and (**b**) diastolic blood pressure (DBP) at 12 weeks and 24 weeks. Values are presented as mean ± standard deviation (SD). Paired t-test; *P* < 0.05 significance; *P < 0.001 compared to baseline

**Fig. 3:**
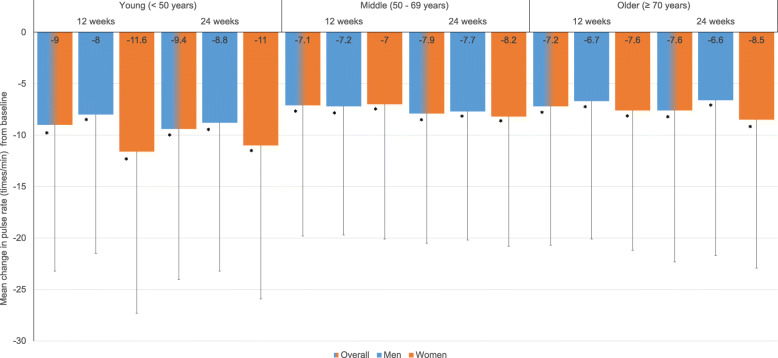
Mean change from baseline in pulse rate after 12 and 24 weeks; Values are presented as mean ± standard deviation; Paired t-test; *P* < 0.05 significance; *P < 0.001 compared to baseline

There was a significant decrease in mean SBP and DBP from baseline at 12 and 24 weeks in both males and females and across all age groups analyzed (all *P* < 0.001 vs. baseline) (Fig. 2a, b). Within the age groups, there was no significant difference in mean reduction in SBP and DBP from baseline between sexes. Similarly, a significant decrease in mean pulse rate from baseline was observed at 12 and 24 weeks in males and females across all age groups analyzed (all *P* < 0.001 vs. baseline) (Fig. 3).

Dizziness, headache, chest pain/discomfort, and dyspnea were the most frequently reported adverse events (Table S1). Majority of the reported adverse events were mild. The incidence of adverse events and adverse drug reactions was lower in young males and females participants compared with middle-aged and older participants (Table 4, Table S2).

**Table 4:** AEs and ADRs in the safety population in BENEFIT-Korea study

Age (yr)	Male		Female	
	AE	ADR	AE	ADR
Young, < 50	26 (7.2)	2 (0.6)	18 (14.5)	3 (2.4)
Middle-aged, 50–69	125 (12.8)	10 (1.0)	83 (16.3)	8 (1.6)
Older, ≥70	84 (15.7)	6 (1.1)	115 (18.1)	7 (1.1)

Values are presented as number (%)

*AE* adverse event, *ADR* adverse drug reaction

## Discussion

In this subanalysis of the efficacy and safety of nebivolol across age and sex in the BENEFIT-KOREA cohort, we observed a significant decrease in the mean SBP and DBP from baseline in both sexes and across all age groups of participants with essential hypertension, with nebivolol demonstrating an acceptable safety and tolerability profile.

Beta-blockers are considered suitable for the treatment of essential hypertension and are currently recommended for the treatment of hypertension, even in older patients, by the ESC/ESH guidelines [5]. Similar to other classes of anti-hypertensives, beta-blockers have been shown to significantly reduce the risk of stroke, heart failure and major cardiovascular events in younger and older patients with hypertension, although their effectiveness has been shown to be age-dependent [20]. Nebivolol is a third-generation vasodilatory β1-adrenergic receptor antagonist which induces nitric oxide-mediated vasodilatory effects via β3 receptor agonism and has demonstrated similar or better treatment response and BP control compared with other anti-hypertensives or their combinations, with significantly better tolerability [21, 22].

Studies evaluating age and sex differences in the efficacy and safety of nebivolol in patients with hypertension are scarce. A retrospective analysis of three randomized controlled trials found that nebivolol monotherapy is efficacious and well tolerated across age groups; however, the efficacy in reducing SBP diminished in patients > 62 years of age [23]. In another study in patients with type 2 diabetes mellitus and hypertension, nebivolol significantly reduced SBP and DBP to a similar extent in both males and females, with a decreasing effect seen with advancing age [24]. In our subanalysis of the BENEFIT-KOREA cohort, nebivolol demonstrated similar efficacy across sex and age in terms of reducing SBP, DBP, and pulse rate in Korean patients with essential hypertension.

Increasing age is associated with increasing prevalence of hypertension in females, particularly in the sixth decade and beyond [4, 8, 9]. We observed an increasing prevalence of hypertension with age in females in the BENEFIT-KOREA cohort, with the highest prevalence seen the older age group (≥70 years). Several factors have been suggested to be associated with the age and sex differences in the BP levels, including sympathetic tone [15]. It can be hypothesized that the effect of beta-blockers on sympathetic tone is mediated by a direct action on presynaptic β-receptors [25]. A study comparing the effects of nebivolol versus atenolol on autonomic function concluded that nebivolol attenuates sympathetic tone similar to atenolol, with the increased bioavailability of nitric oxide by nebivolol serving as a stimulus to inhibit sympathetic outflow [26, 27]. In our subanalysis, nebivolol, a vasodilatory β1-adrenergic receptor antagonist, was found to be safe and efficacious in all patients with hypertension with or without comorbidities, regardless of age and sex.

We observed that the total dose of nebivolol administered to the study participants was significantly lower in females and in older patients. Despite sex differences in hypertension, the treatment guidelines for hypertension do not differ by sex [3]. However, in Korea, physicians tend to prescribe lower dose of medications to females compared with males, potentially to account for the differences in body weight. Older patients are also prescribed lower doses of nebivolol in clinical practice versus younger patients to account for potential renal impairment issues with advancing age (even in the absence of disease) which could lead to reduced drug clearance in turn leading to an effect of equivalent drug concentration despite the lower dose [28, 29].

This analysis is based on an observational cohort. Also, a majority of the patients in this subanalysis were ≥ 50 years of age, so there is a limited generalizability of the study findings to a younger group of patients (< 50 years). Despite these limitations, we believe that our subanalysis demonstrated the efficacy and safety of once-daily nebivolol across age groups with no between-sex differences.

## Conclusions

Our subanalysis from the real-world BENEFIT-KOREA study shows that nebivolol demonstrated similar efficacy across sex and age in terms of reducing SBP, DBP and pulse rate in Korean patients with essential hypertension.

The prevalence of hypertension is known to differ by age and sex. Nebivolol has been reported to be efficacious and well tolerated for achieving BP control in patients with hypertension in randomized studies as well real-world observational studies [16, 17]. However, studies investigating age and sex differences in the efficacy and safety of nebivolol in patients with hypertension are scarce – our age and sex-based subanalysis from the large, observational BENEFIT-KOREA study provides further evidence to support clinicians managing patients with hypertension.

## Supplementary Information

**Additional file 1: Table S1.** Adverse events (AEs) with an incidence of ≥0.5% in at least one of the subgroups in the safety population in the BENEFIT-KOREA study. **Table S2.** Adverse drug reactions (ADRs) in the safety population in BENEFIT-KOREA study.

**Additional file 2.** list of IRB site.

## Data Availability

The datasets used and/or analyzed during the current study are available from the corresponding author on reasonable request.

## Abbreviations

ACE: Angiotensin-converting enzyme
ADR: adverse drug reaction
AE: Adverse event
BENEFIT-KOREA: BEnefits after 24 weeks of NEbivolol administration For essential hypertensIon patients wiTh various comorbidities and treatment environments in Korea
BMI: Body mass index
BP: Blood pressure
DBP: Diastolic blood pressure
ESC/ESH: European Society of Cardiology/European Society of Hypertension
KNHANES: Korean National Health and Nutrition Examination Survey
MedDRA: Medical Dictionary for Regulatory Activities
SBP: Systolic blood pressure
SD: Standard deviation
