# Supplementary material for: Efficacy and safety of nebivolol in Korean patients with hypertension by age and sex: a subanalysis from the BENEFIT-KOREA study
Source: Clin Hypertens. 2021 Mar 15;27:9. doi: 10.1186/s40885-021-00165-3 (PMC7958700; doi:10.1186/s40885-021-00165-3)
Supplement: Supplementary file 1 — Additional file 1: Table S1. Adverse events (AEs) with an incidence of ≥0.5% in at least one of the subgroups in the safety population in the BENEFIT-KOREA study. Table S2. Adverse drug reactions (ADRs) in the safety population in BENEFIT-KOREA study. [file 40885_2021_165_MOESM1_ESM.docx]

**Title: Efficacy and safety of nebivolol in Korean patients with hypertension by age and sex: a subanalysis from the BENEFIT-KOREA study**

**Supplementary Table 1.** Adverse events with an incidence of ≥0.5% in at least one of the subgroups in the safety population in the BENEFIT-KOREA study

| Variable | Incidence | | | | | |
| --- | --- | --- | --- | --- | --- | --- |
|  | **Young (< 50 yr)** | | **Middle (50−69 yr)** | | **Older (≥70 yr)** | |
| Description^a)^ | Male  (n=359) | Female  (n=124) | Male  (n=976) | Female  (n=508) | Male  (n=536) | Female  (n=637) |
| Adverse event |  |  |  |  |  |  |
| Dizziness | 2 (0.6) | 5 (4.0) | 5 (0.5) | 12 (2.4) | 10 (1.9) | 8 (1.3) |
| Headache | - | 3 (2.4) | 5 (0.5) | 11 (2.2) | 4 (0.7) | 8 (1.3) |
| Paraesthesia | 2 (0.6) | - | - | 2 (0.4) | 2 (0.4) | 1 (0.2) |
| Chest pain | 1 (0.3) | - | 12 (1.2) | 3 (0.6) | 4 (0.7) | 3 (0.5) |
| Chest discomfort | 2 (0.6) | - | 5 (0.5) | 4 (0.8) | 5 (0.9) | 2 (0.3) |
| Face oedema | - | - | - | 2 (0.4) | - | 3 (0.5) |
| Oedema peripheral | - | - | - | - | 1 (0.2) | 3 (0.5) |
| Cough | - | 1 (0.8) | - | 1 (0.2) | 2 (0.4) | 3 (0.5) |
| Dyspnea | - | - | 4 (0.4) | 4 (0.8) | 4 (0.7) | 17 (2.7) |
| Dyspnea exertional | - | - | 7 (0.7) | - | 1 (0.2) | 3 (0.5) |
| Epistaxis | - | - | - | 1 (0.2) | 3 (0.6) | 1 (0.2) |
| Gastritis | - | - | - | 1 (0.2) | 1 (0.2) | 4 (0.6) |
| Nausea | - | - | 2 (0.2) | - | - | 3 (0.5) |
| Gastroesophageal reflux disease | - | 1 (0.8) | - | 1 (0.2) | - | 2 (0.3) |
| Dyspepsia | 1 (0.3) | - | 2 (0.2) | 4 (0.8) | 2 (0.4) | 1 (0.2) |
| Pain in extremity | - | 1 (0.8) | 1 (0.1) | 1 (0.2) | - | 2 (0.3) |
| Myalgia | - | - | - | 3 (0.6) | - | - |
| Palpitations | 1 (0.3) | 1 (0.8) | 2 (0.2) | 4 (0.8) | 3 (0.6) | 1 (0.2) |
| Urinary tract infection | - | - | - | 1 (0.2) | - | 3 (0.5) |
| Viral upper respiratory tract infection | - | 1 (0.8) | - | 1 (0.2) | - | - |
| Hyperlipidemia | 2 (0.6) | - | 4 (0.4) | 1 (0.2) | - | - |
| Dry eye | - | - | 1 (0.1) | - | 2 (0.4) | 3 (0.5) |
| Retinopathy hypertensive | - | 1 (0.8) | - | - | - | - |
| Pruritus | - | - | - | - | 1 (0.2) | 4 (0.6) |
| Contusion | 2 (0.6) | - | 1 (0.1) | - | - | - |
| Uterine leiomyoma | - | 1 (0.8) | - | - | - | - |
| Anemia | - | 1 (0.8) | - | 1 (0.2) | - | - |
| Nystagmus | - | 1 (0.8) | - | - | - | - |
| Condition aggravated | - | 1 (0.8) | - | - | - | - |
| Intervertebral disc protrusion | - | 1 (0.8) | - | - | - | - |

Values are presented as number (%).

^a)^Medical Dictionary for Regulatory Activities (MedDRA) 20.0.

**Supplementary Table 2.** Adverse drug reactions in the safety population in BENEFIT-KOREA study

| Variable | Incidence | | | | | |
| --- | --- | --- | --- | --- | --- | --- |
|  | **Young (< 50 yr)** | | **Middle (50−69 yr)** | | **Older (≥70 yr)** | |
| Description^a)^ | Male  (359) | Female  (124) | Male  (976) | Female  (508) | Male  (536) | Female  (637) |
| Any adverse drug reaction | 2 (0.6) | 3 (2.4) | 13 (1.3) | 8 (1.6) | 6 (1.1) | 7 (1.1) |
| Paraesthesia | 1 (0.28) | - | - | 2 (0.4) | - | - |
| Erectile dysfunction | 1 (0.28) | - | - | - | - | - |
| Dizziness | - | 3 (2.4) | - | 2 (0.4) | - | 1 (0.2) |
| Headache | - | - | - | 2 (0.4) | - | - |
| Hypoaesthesia | - | - | 1 (0.1) | - | - | - |
| Syncope | - | - | 1 (0.1) | - | - | - |
| Dizziness postural | - | - | 1 (0.1) | - | - | - |
| Burning sensation | - | - | 1 (0.1) | - | - | - |
| Lethargy | - | - | 1 (0.1) | - | - | - |
| Chest pain | - | - | 1 (0.1) | - | - | - |
| Asthenia | - | - | - | - | 1 (0.2) | - |
| Face edema | - | - | - | - | - | 1 (0.2) |
| Edema | - | - | 1 (0.1) | - | - | - |
| Dyspnea | - | - | 1 (0.1) | - | 2 (0.4) | - |
| Dyspnea exertional | - | - | 1 (0.1) | - | - | - |
| Dry mouth | - | - | - | - | 1 (0.2) | - |
| Arthralgia | - | - | 1 (0.1) | - | - | - |
| Bradycardia | - | - | 1 (0.1) | 2 (0.4) | 1 (0.2) | 2 (0.3) |
| Pruritus | - | - | - | - | - | 1 (0.2) |
| Blood pressure decreased | - | - | 1 (0.1) | 1 (0.2) | - | - |
| Blood pressure increased | - | - | - | 1 (0.2) | - | - |
| Heart rate decreased | - | - | - | - | 1 (0.2) | 1 (0.2) |
| Orthostatic hypotension | - | - | 1 (0.1) | - | - | - |
| Hypotension | - | - | - | - | - | 1 (0.2) |

Values are presented as number (%).

^a)^Medical Dictionary for Regulatory Activities (MedDRA) 20.0.
